# Omadacycline dihydrate, C_29_H_40_N_4_O_7_·2H_2_O, from X-ray powder diffraction data

**DOI:** 10.1107/S2056989024001403

**Published:** 2024-02-16

**Authors:** James A. Kaduk, Nicholas C. Boaz, Stacy Gates-Rector, Amy M. Gindhart, Thomas N. Blanton

**Affiliations:** a ICDD, 12 Campus Blvd., Newtown Square PA 19073-3273, USA; b Department of Chemistry, North Central College, 131 S. Loomis, St., Naperville IL, 60540 , USA; University of Aberdeen, United Kingdom

**Keywords:** powder diffraction, omadacycline, Nuzyra, Rietveld refinement

## Abstract

The crystal structure of the title compound {systematic name: (4*S*,4a*S*,5a*R*,12a*R*)-4,7-bis­(di­methyl­amino)-9-[(2,2-di­methyl­propyl­amino)­meth­yl]-1,10,11,12a-tetra­hydroxy-3,12-dioxo-4a,5,5a,6-tetra­hydro-4*H*-tetra­cene-2-carb­oxamide dihydrate, C_29_H_40_N_4_O_7_·2H_2_O} has been solved and refined using synchrotron X-ray powder diffraction data: it crystallizes in space group *R*3 with *a* = 24.34430 (7), *c* = 14.55212 (4) Å, *V* = 7468.81 (2) Å^3^ and *Z* = 9. Most of the hydrogen bonds are intra­molecular, but two classical N—H⋯O inter­molecular hydrogen bonds (along with probable weak C—H⋯O and C—H⋯N hydrogen bonds) link the mol­ecules into a three-dimensional framework. The framework contains voids, which contain disordered water mol­ecules. Keto–enol tautomerism is apparently important in this mol­ecule, and the exact mol­ecular structure is ambiguous.

## Abstract 2

The crystal structure of omadacycline dihydrate has been solved and refined using synchrotron X-ray powder diffraction data.

## 1. Chemical context

Omadacycline, sold under the brand name Nuzyra, is a broad-spectrum tetra­cycline anti­biotic. Omadacycline finds use in treating bacterial pneumonia and certain types of skin infections. The systematic name (CAS Registry No. 389139-89-3) is (4*S*,4a*S*,5a*R*,12a*R*)-4,7-bis­(di­methyl­amino)-9-[(2,2-di­methyl­propyl­amino)­meth­yl]-1,10,11,12a-tetra­hydroxy-3,12-dioxo-4a,5,5a,6-tetra­hydro-4H-tetra­cene-2-carboxamide. It is sometimes the case that the hydroxyl and carbonyl groups are misassigned in structure pictures of tetra­cycline anti­biotics, so in addition to the crystal structure it is important to consider the chemical connectivity to give insight into keto–enol tautomerism. 
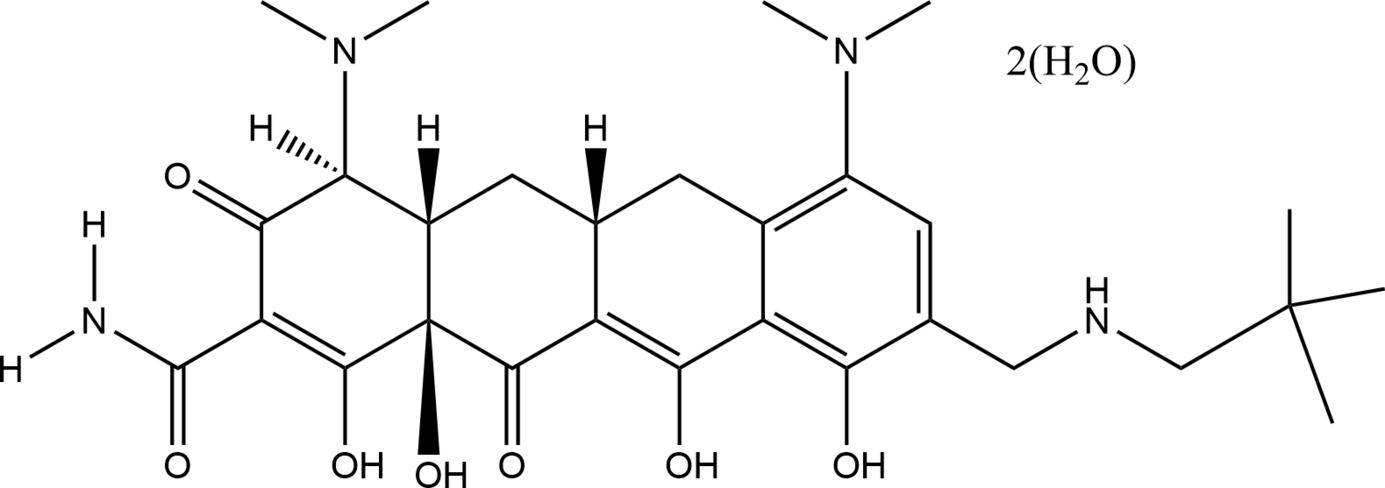


This work was carried out as part of a project (Kaduk *et al.*, 2014 ▸) to determine the crystal structures of large-volume commercial pharmaceuticals, and include high-quality powder diffraction data for them in the Powder Diffraction File (Gates-Rector & Blanton, 2019 ▸).

## 2. Structural commentary

**Figure 1:**
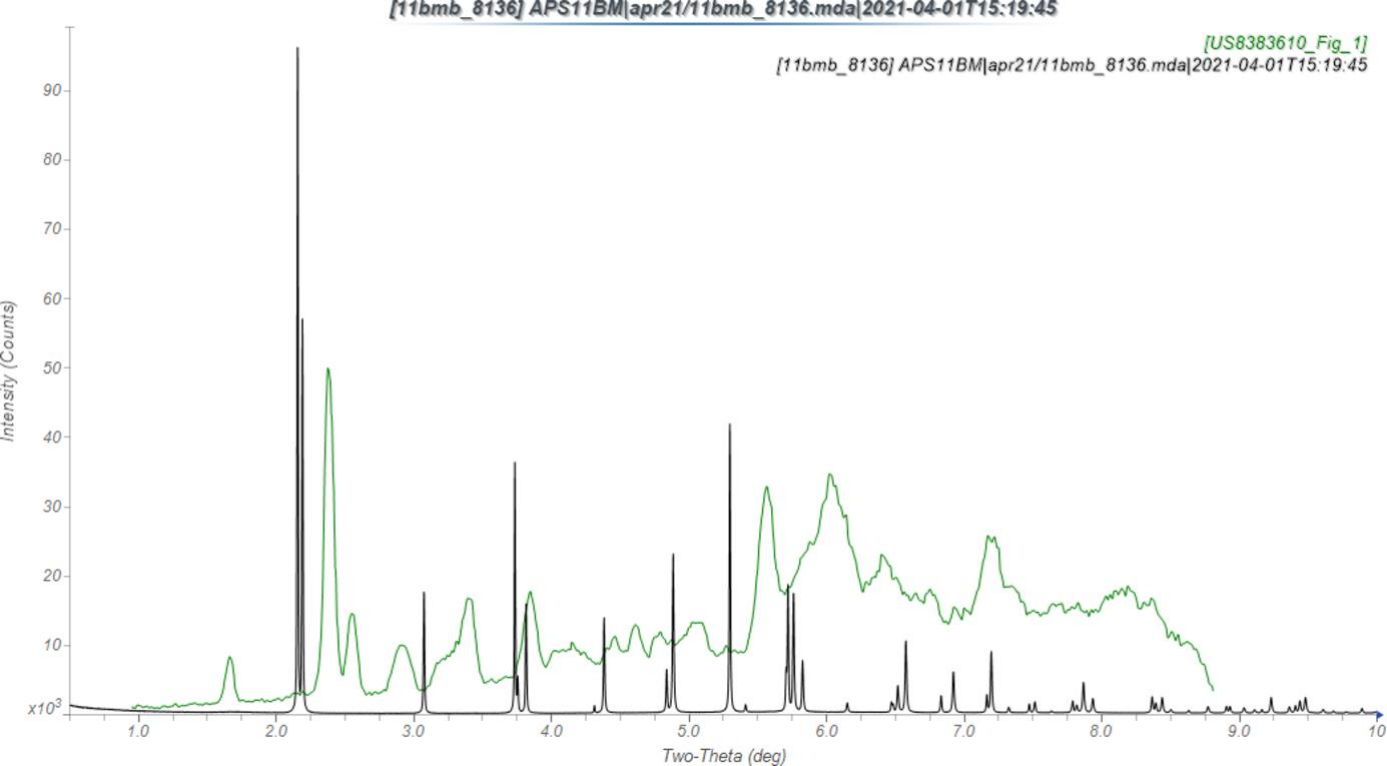
Comparison of the synchrotron pattern of omadacycline dihydrate (black) to that of omadacycline (green) reported by Cvetovich & Warchol (2013 ▸). The patent pattern, measured using Cu *K*α radiation, was digitized using *UN-SCAN-IT* (Silk Scientific, 2013 ▸), and converted to the synchrotron wavelength of 0.458133 Å using *JADE Pro* (MDI, 2021 ▸). Image generated using *JADE Pro* (MDI, 2021 ▸).

The powder pattern of the hydrated omadacycline studied here is not the same as that reported for crystalline omadacycline by Cvetovich & Warchol (2013 ▸) (Fig. 1 ▸). Our material was a commercial sample, but it is not clear how representative it is of the bulk pharmaceutical material.

**Figure 2:**
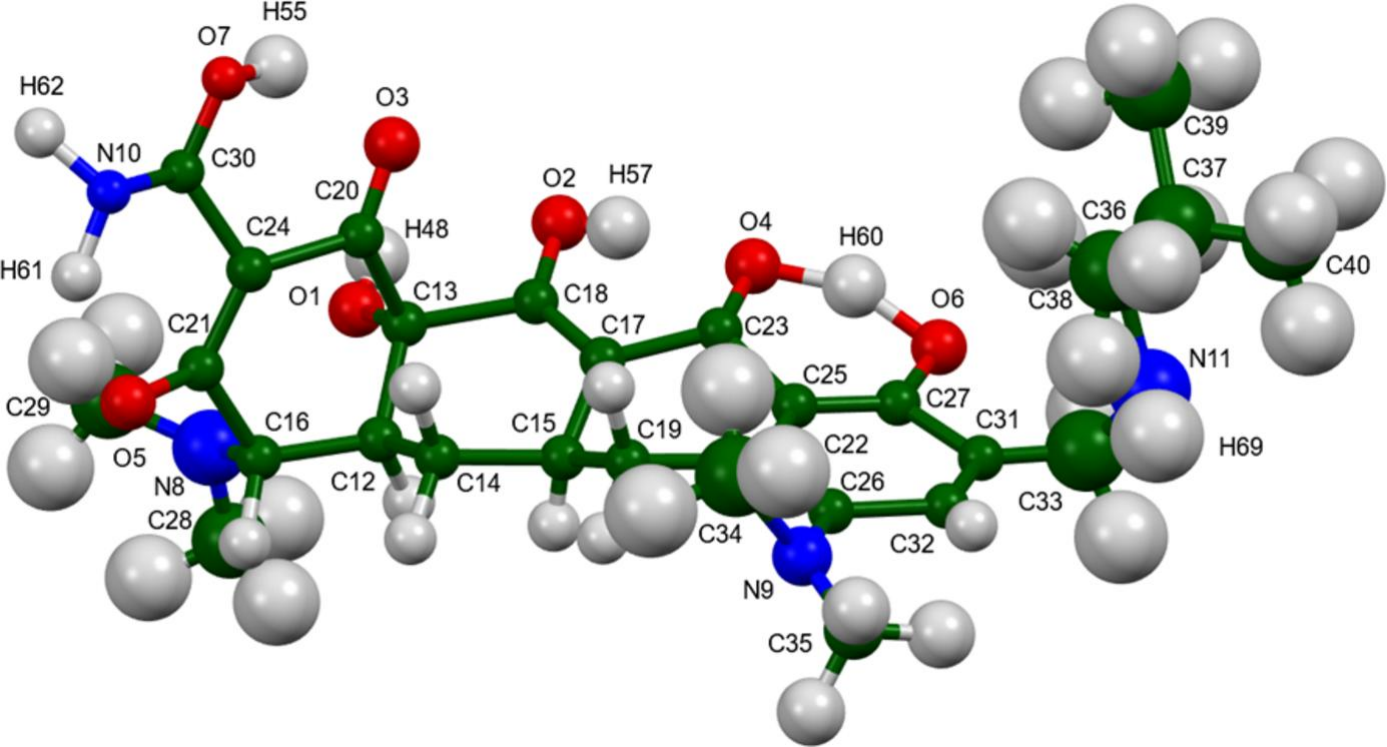
The omadacycline mol­ecule in omadacycline dihydrate, with the atom numbering. The atoms are represented by 50% probability spheres.

The refined structure of the omadacycline mol­ecule is different in chemical connectivity and conformation from that archived in PubChem (Kim *et al.*, 2019 ▸; Figs. 2 ▸ and 3 ▸). In particular, C20—O3 (our numbering scheme) is a double bond, while C30—O7, C21—O5, and C18—O2 are single bonds. C21—C24 is a double bond, C20—C24 is a single bond, and several other C—C bonds in the ring system differ in order. It is unclear whether the differences represent differences between solution and the solid state, or merely the limited information content of the powder diffraction pattern of a very complex material. The bond-distance and bond-angle restraints were deliberately given low weight to gain insight into what information the diffraction data can give regarding the chemical connectivity.

It was clear from both the structure solution and refinement and a DFT calculation that the C30—O7—N10 group is oriented to form a strong intra­molecular O7—H55⋯O3 hydrogen bond, and that N10 participates in inter­molecular hydrogen bonds (Table 1 ▸).

There are many unusual bond distances, bond angles, and torsion angles indicated by a *Mercury* Mogul Geometry check (Macrae *et al.*, 2020 ▸). Although there are some large *Z*-scores among the bond distances, the largest ones tend to be on the periphery of the mol­ecule, where the chemical connectivity is not in doubt. In the ring system, the distinctions between single and double bonds seem to be clear. It is hard to make conclusions about the *Z*-scores of the bond angles, but some of the large *Z*-scores result from very small standard uncertainties on the average bond angles. Both for bond distances and bond angles, greater- and less-than-normal values tend to be correlated, probably reflecting errors in atom positions, which were restrained only modestly. Some torsion angles involving rotation about the C16—N8, C26—N9, C24—C30, C37—C36, and C31—C33 bonds are flagged as unusual. All of these reflect the orientations of peripheral groups, which do seem to be unusual in this crystal structure.

## 3. Supra­molecular features

We obtained guidance on whether potential inter­atomic contacts were real hydrogen bonds from a DFT optimization of the anhydrous structure (without the disordered water mol­ecules). This structure is close to that of the disordered anhydrate. A DFT optimization of an ordered dihydrate yielded a different mol­ecular connectivity, and it is unclear how relevant such a calculation is to the disordered structure. The differences point out that the mol­ecular connectivity may vary depending on the state of hydration, and also in solution *versus* the solid state.

**Figure 4:**
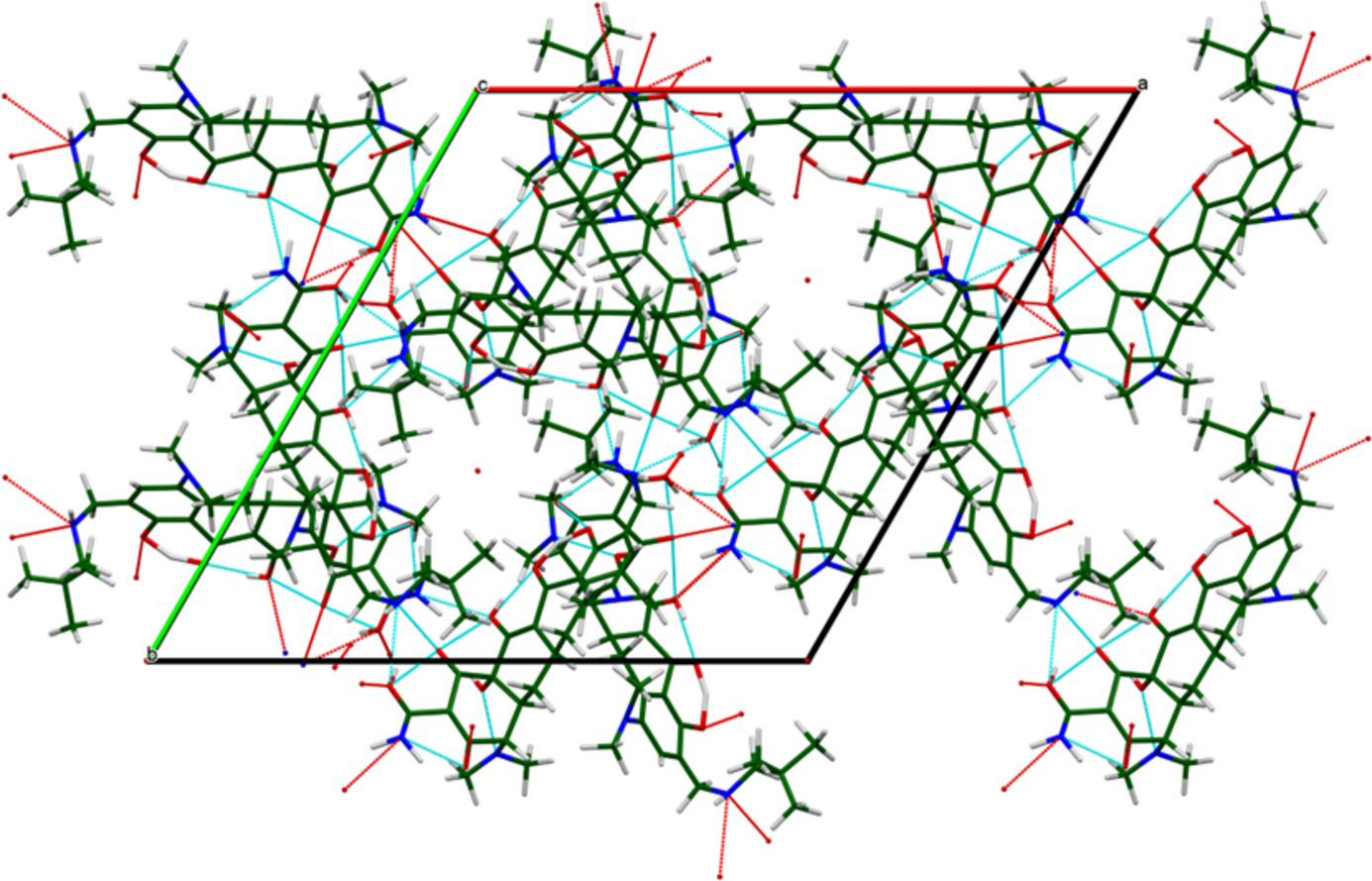
The crystal structure of omadacycline dihydrate, viewed down the *c*-axis direction showing the voids occupied by disordered water mol­ecules.

There are many hydrogen bonds in the structure, but (perhaps surprisingly) almost all of them are intra­molecular. Only the N10—H62⋯O2 and N11—H69⋯O84 hydrogen bonds (as well as probable weak C—H⋯O and C—H⋯N hydrogen bonds) link different mol­ecules. The inter­molecular hydrogen bonds link the mol­ecules into a three-dimensional network (Fig. 4 ▸). There are three very strong intra­molecular O—H⋯O hydrogen bonds between hydroxyl and carbonyl groups. There are also short intra­molecular meth­yl⋯methyl contacts between H49 and H52 and H66 and H63. The shortest (and only) O⋯O distances between water mol­ecules and omadacycline mol­ecules are 2.727 (17) and 3.119 (16) Å between O84 and two symmetry-equivalent O7; the water mol­ecules only loosely inter­act with the framework and it was not possible to unambiguously locate the water H atoms.

We may state that we have established the *crystal* structure of omadacyclic dihydrate, but the exact mol­ecular structure is ambiguous.

## 4. Database survey

Polymorphs of crystalline omadacycline tosyl­ate are claimed in US Patent 8,383,610 B2 (Cvetovich & Warchol, 2013 ▸; Paratek Pharmaceuticals). A powder pattern of the parent compound is also provided. A reduced cell search in the Cambridge Structural Database (CSD, version 5.45 November 2023; Groom *et al.*, 2016 ▸) combined with C, H, N, and O only, yielded two hits, but no structures of omadacycline derivatives.

## 5. Synthesis and crystallization

Omadacycline was a commercial reagent, purchased from TargetMol (Batch #132019), and was used as received.

## 6. Refinement

Crystal data, data collection and structure refinement details are summarized in Table 2 ▸.

The pattern was first indexed using *JADE Pro 8.1* (MDI, 2021 ▸) as a primitive monoclinic unit cell with *a* = 11.98344, *b* = 12.17479, *c* = 8.54255 Å, *β* = 91.30°, *V* = 1246.0 Å^3^, and *Z* = 2. Indexing using *N-TREOR* (Altomare *et al.*, 2013 ▸) yielded a hexa­gonal unit cell with *a* = 24.34510, *c* = 14.55468 Å, and *V* = 7470.6 Å^3^. Re-indexing with *JADE*, allowing for higher-symmetry cells, yielded the same hexa­gonal cell. The space group suggested by both programs was *R*3.

An omadacycline mol­ecule was downloaded from PubChem (Kim *et al.*, 2019 ▸) as Conformer3D_CID_5469735.sdf. It was converted into a *.mol2 file using *Mercury* (Macrae *et al.*, 2020 ▸), and into a Fenske-Hall *Z*-matrix file using *OpenBabel* (O’Boyle *et al.*, 2011 ▸). The structure was solved using *FOX* (Favre-Nicolin & Černý, 2002 ▸) using (sin θ/λ)_max_ = 0.4 Å^−1^. Visualization of the structure revealed the presence of several voids. By placing oxygen atoms (possibly water mol­ecules) into the voids and refining their positions and occupancies (some refined to less than 0, and were removed from the model), four potential sites, corresponding to 18.1 H_2_O/cell, or 2.0 H_2_O/omadacycline (*i.e.*, a dihydrate) were identified.

NMR analysis of the omadacycline sample was performed using a 400 MHz Bruker Avance spectrometer equipped with a multinuclear probe. The ^1^H NMR of the pharmaceutical sample was performed in *d* ^6^ DMSO, which was stored over flame-dried 3 Å mol­ecular sieves. The ^1^H NMR analysis of the sample indicated the presence of water in addition to omadacycline (Gottlieb *et al.*, 1997 ▸). By comparing the water signal at 3.33 ppm to the signal at 7.41 ppm, which belongs to the arene C—H group of the pharmaceutical moleucle, the water content was estimated to be approximately 1.5 water mol­ecules to 1 omadacycline. Moreover, the ^1^H NMR spectrum of the omadacycline sample indicated that there was no observable trace of residual organic solvent or tosyl­ate counter-ion. The NMR data therefore indicate that the species in the pores in the crystal structure is water. After evaporation of the DMSO solvent, the solid was discolored.

**Figure 5:**
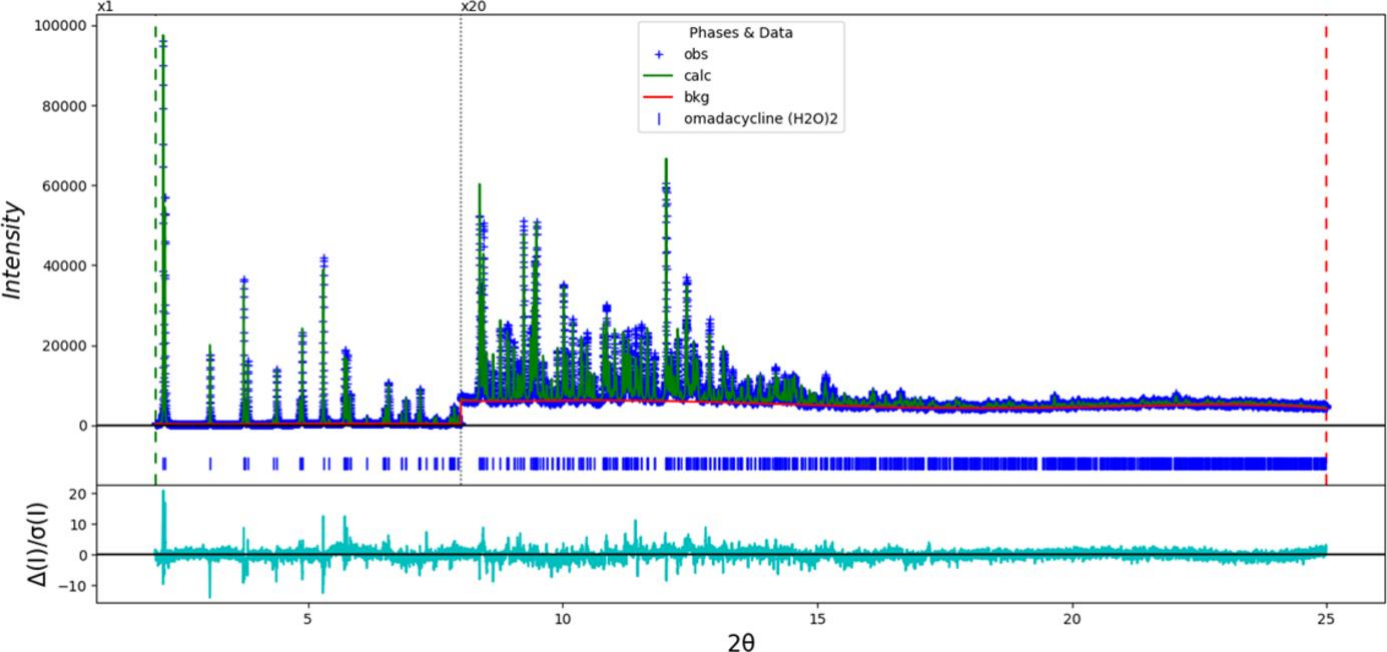
The Rietveld plot for the refinement of omadacycline dihydrate. The blue crosses represent the observed data points, and the green line is the calculated pattern. The cyan curve is the normalized difference plot. The vertical scale has been multiplied by a factor of 20× for 2θ > 8.0°.

Rietveld refinement (Fig. 5 ▸) was carried out using *GSAS-II* (Toby & Von Dreele, 2013 ▸). Only the 2.0–25.0° portion of the pattern was included in the refinement (*d*_min_ = 1.058 Å). The *z*-coordinate of O1 was fixed to define the origin. All non-H bond distances and angles were subjected to restraints, based on a *Mercury* Mogul Geometry Check (Sykes *et al.*, 2011 ▸; Bruno *et al.*, 2004 ▸). The Mogul average and standard deviation for each qu­antity were used as the restraint parameters. The weight of the restraints was gradually decreased during the refinement. The restraints contributed 3.8% to the final χ^2^. The hydrogen atoms were included in calculated positions, which were recalculated during the refinement using *Materials Studio* (Dassault, 2021 ▸). The *U_iso_* values were grouped by chemical similarity. The *U*_iso_ for the H atoms were fixed at 1.3 × the *U*_iso_ of the heavy atoms to which they are attached. A second-order spherical harmonic model was included in the refinement to account for preferred orientation and the refined texture index is 1.001 (0). The peak profiles were described using the generalized microstrain model. The background was modeled using a six-term shifted Chebyshev polynomial, plus a peak at 5.63° 2θ to model the scattering from the Kapton capillary and any amorphous component.

## Supplementary Material

Crystal structure: contains datablock(s) I. DOI: 10.1107/S2056989024001403/hb8082sup1.cif

Supporting information file. DOI: 10.1107/S2056989024001403/hb8082Isup2.cml

CCDC reference: 2332637

Additional supporting information:  crystallographic information; 3D view; checkCIF report

## Figures and Tables

**Figure 3:**
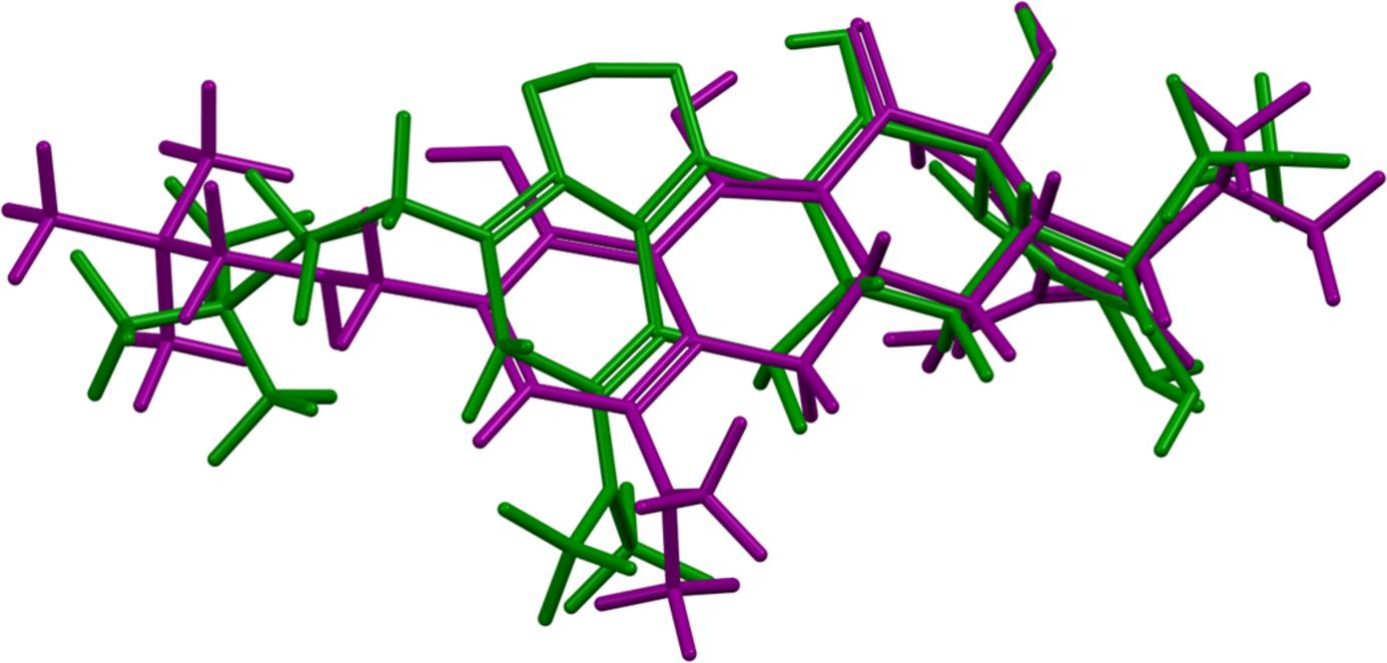
Comparison of the structure of the omadacycline mol­ecule from this Rietveld refinement (green) to that archived in PubChem (purple). The root-mean-square Cartesian displacement of the non-H atoms is 1.08 Å.

**Table 1:** Hydrogen-bond geometry (Å, °)

*D*—H⋯*A*	*D*—H	H⋯*A*	*D*⋯*A*	*D*—H⋯*A*
O7—H55⋯O3	0.98	1.84	2.614 (15)	134
O2—H57⋯O4	0.74	1.78	2.506 (15)	169
O6—H60⋯O4	1.28	1.26	2.485 (15)	155
O1—H48⋯O2	0.82	2.42	2.721 (13)	103
N10—H61⋯O5	1.11	1.79	2.686 (12)	135
N10—H62⋯O2^i^	1.11	2.04	2.935 (13)	135
N11—H69⋯O84^ii^	1.11	2.15	3.18 (2)	153
C33—H58⋯N10^iii^	1.14	2.51	3.63 (2)	165
C34—H64⋯O1^iv^	1.14	2.35	3.46 (2)	165
C35—H68⋯O1^iv^	1.14	2.58	3.642 (15)	154
C39—H76⋯O85^v^	1.18	2.41	3.32 (2)	132

Symmetry codes: (i) 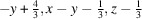 ; (ii) 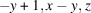 ; (iii) 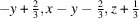 ; (iv) 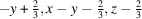 ; (v) 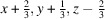 .

**Table 2:** Experimental details

Crystal data	
Chemical formula	C_29_H_40_N_4_O_7_·2H_2_O
*M*_r_	588.03
Crystal system, space group	Trigonal, *R*3
Temperature (K)	295
*a*, *c* (Å)	24.34430 (7), 14.55212 (4)
*V* (Å^3^)	7468.81 (2)
*Z*	9
Radiation type	Synchrotron, λ = 0.45813 Å
μ (mm^−1^)	0.01
Specimen shape, size (mm)	Cylinder, 3 × 1.5

Data collection	
Diffractometer	11-BM, APS
Specimen mounting	Kapton capillary
Data collection mode	Transmission
Scan method	Step
2θ values (°)	2θ_min_ = 0.500, 2θ_max_ = 49.997, 2θ_step_ = 0.001

Refinement	
*R* factors and goodness of fit	*R*_p_ = 0.048, *R*_wp_ = 0.061, *R*_exp_ = 0.043, *R*(*F* ^2^) = 0.06407, χ^2^ = 2.161
No. of parameters	148
No. of restraints	112
H-atom treatment	Only H-atom displacement parameters refined
(Δ/σ)_max_	4.723

Computer programs: *GSAS-II* (Toby & Von Dreele, 2013 ▸), *Mercury* (Macrae *et al.*, 2020 ▸), and *publCIF* (Westrip, 2010 ▸).

## supplementary crystallographic information

### Crystal data

**Table:**

C_29_H_40_N_4_O_7_·2H_2_O	*Z* = 9
*M**_r_* = 588.03	*D*_x_ = 1.177 Mg m^−^^3^
Trigonal, *R*3	Synchrotron radiation, λ = 0.45813 Å
Hall symbol: R 3	µ = 0.01 mm^−^^1^
*a* = 24.34430 (7) Å	*T* = 295 K
*c* = 14.55212 (4) Å	white
*V* = 7468.81 (2) Å^3^	cylinder, 3 × 1.5 mm

### Data collection

**Table:**

11-BM, APS diffractometer	Data collection mode: transmission
Radiation source: synchrotron	Scan method: step
Double Si(111) sngle crystal monochromator	2θ_min_ = 0.500°, 2θ_max_ = 49.997°, 2θ_step_ = 0.001°
Specimen mounting: Kapton capillary	

### Refinement

**Table:**

Least-squares matrix: full	148 parameters
*R*_p_ = 0.048	112 restraints
*R*_wp_ = 0.061	7 constraints
*R*_exp_ = 0.043	Only H-atom displacement parameters refined
*R*(*F*^2^) = 0.06407	Weighting scheme based on measured s.u.'s
49575 data points	(Δ/σ)_max_ = 4.723
Excluded region(s): Th regions 0.5-2.0 and 25.0-50.0° contained no peaks.	Background function: Background function: "chebyschev-1" function with 6 terms: 242.80(28), -5.7(4), -17.4(4), 6.6(4), 19.4(3), -39.10(30), Background peak parameters: pos, int, sig, gam: 5.635, 59368.501, 16287.514, 0.100,
Profile function: Finger-Cox-Jephcoat function parameters U, V, W, X, Y, SH/L: peak variance(Gauss) = Utan(Th)^2^+Vtan(Th)+W: peak HW(Lorentz) = X/cos(Th)+Ytan(Th); SH/L = S/L+H/L U, V, W in (centideg)^2^, X & Y in centideg 1.163, -0.126, 0.063, 0.000, 0.000, 0.002,	Preferred orientation correction: Simple spherical harmonic correction Order = 2 Coefficients: 0:0:C(2,0) = 0.081(5)

### Fractional atomic coordinates and isotropic or equivalent isotropic displacement parameters (Å^2^)

**Table:**

	*x*	*y*	*z*	*U*_iso_*/*U*_eq_	Occ. (<1)
O1	0.8514 (3)	0.1354 (3)	0.51110	0.0497 (10)*	
O2	0.7746 (4)	0.1858 (4)	0.4890 (7)	0.0497 (10)*	
O3	0.8837 (4)	0.2363 (4)	0.3587 (8)	0.0497 (10)*	
O4	0.6658 (4)	0.1617 (4)	0.4417 (7)	0.0497 (10)*	
O5	0.9444 (4)	0.0918 (4)	0.2428 (7)	0.0497 (10)*	
O6	0.5495 (4)	0.1148 (4)	0.4214 (7)	0.0497 (10)*	
O7	0.9888 (4)	0.2785 (4)	0.2650 (7)	0.031 (2)*	
N8	0.8862 (7)	0.0578 (6)	0.4539 (9)	0.092 (4)*	
N9	0.5642 (5)	0.0056 (5)	0.1009 (9)	0.057 (3)*	
N10	1.0152 (5)	0.2154 (4)	0.2006 (9)	0.031 (2)*	
N11	0.4343 (6)	0.0952 (7)	0.2570 (10)	0.107 (3)*	
C12	0.8049 (5)	0.0645 (5)	0.3856 (9)	0.0336 (9)*	
C13	0.8248 (4)	0.1300 (5)	0.4225 (7)	0.0336 (9)*	
C14	0.7724 (5)	0.0573 (5)	0.2953 (9)	0.0336 (9)*	
C15	0.7089 (5)	0.0561 (6)	0.3079 (9)	0.0336 (9)*	
C16	0.8606 (5)	0.0550 (6)	0.3636 (10)	0.0336 (9)*	
C17	0.7161 (5)	0.1108 (5)	0.3646 (9)	0.0336 (9)*	
C18	0.7652 (5)	0.1424 (5)	0.4282 (9)	0.0336 (9)*	
C19	0.6766 (5)	0.0564 (6)	0.2273 (9)	0.0336 (9)*	
C20	0.8734 (5)	0.1842 (5)	0.3436 (9)	0.0336 (9)*	
C21	0.9163 (6)	0.1090 (5)	0.3060 (8)	0.0336 (9)*	
C22	0.6082 (4)	0.0559 (6)	0.2353 (10)	0.0336 (9)*	
C23	0.6654 (5)	0.1270 (5)	0.3751 (8)	0.0336 (9)*	
C24	0.9206 (5)	0.1664 (5)	0.3003 (9)	0.0336 (9)*	
C25	0.6071 (4)	0.0848 (5)	0.3199 (9)	0.0336 (9)*	
C26	0.5612 (5)	0.0287 (5)	0.1824 (9)	0.0336 (9)*	
C27	0.5555 (6)	0.0851 (6)	0.3527 (8)	0.0336 (9)*	
C28	0.8490 (8)	0.0023 (8)	0.5002 (10)	0.092 (4)*	
C29	0.9504 (7)	0.0844 (7)	0.4781 (12)	0.092 (4)*	
C30	0.9828 (5)	0.2244 (5)	0.2738 (10)	0.031 (2)*	
C31	0.4989 (6)	0.0548 (6)	0.2996 (9)	0.0336 (9)*	
C32	0.5042 (6)	0.0304 (6)	0.2106 (9)	0.0336 (9)*	
C33	0.4478 (7)	0.0581 (7)	0.3171 (11)	0.107 (3)*	
C34	0.6218 (7)	0.0548 (7)	0.0311 (13)	0.107 (3)*	
C35	0.5180 (7)	−0.0357 (7)	0.0567 (9)	0.057 (3)*	
C36	0.4804 (6)	0.1595 (6)	0.2292 (10)	0.057 (3)*	
C37	0.4713 (8)	0.1946 (8)	0.1400 (11)	0.107 (3)*	
C38	0.4917 (7)	0.1708 (7)	0.0725 (13)	0.107 (3)*	
C39	0.5218 (8)	0.2717 (8)	0.1705 (10)	0.107 (3)*	
C40	0.4133 (8)	0.1868 (7)	0.1118 (13)	0.107 (3)*	
H41	0.77134	0.02666	0.43592	0.0437 (11)*	
H42	0.76306	0.01119	0.26141	0.0437 (11)*	
H43	0.80423	0.09882	0.24866	0.0437 (11)*	
H44	0.69353	0.01603	0.32895	0.0437 (11)*	
H45	0.84361	0.00660	0.33080	0.0437 (11)*	
H46	0.70821	0.10088	0.18560	0.0437 (11)*	
H47	0.66787	0.01369	0.18415	0.0437 (11)*	
H48	0.85543	0.16435	0.54461	0.0646 (13)*	
H49	0.80861	−0.03198	0.45374	0.120 (5)*	
H50	0.87885	−0.01974	0.52155	0.120 (5)*	
H51	0.82856	0.01244	0.56423	0.120 (5)*	
H52	0.95992	0.04520	0.50130	0.120 (5)*	
H53	0.98138	0.10985	0.41609	0.120 (5)*	
H54	0.96198	0.11972	0.53653	0.120 (5)*	
H56	0.48709	0.02113	0.14240	0.0437 (11)*	
H57	0.74402	0.18307	0.47700	0.0646 (13)*	
H58	0.45430	0.07785	0.38980	0.139 (4)*	
H59	0.40539	0.00784	0.31716	0.139 (4)*	
H60	0.60944	0.15005	0.43402	0.0646 (13)*	
H61	1.00708	0.16631	0.20287	0.040 (3)*	
H62	1.06675	0.24970	0.20670	0.040 (3)*	
H63	0.64974	0.10332	0.06510	0.139 (4)*	
H64	0.60037	0.05953	−0.03602	0.139 (4)*	
H65	0.65485	0.03534	0.01626	0.139 (4)*	
H66	0.47237	−0.03954	0.08425	0.074 (4)*	
H67	0.51579	−0.08335	0.06450	0.074 (4)*	
H68	0.52365	−0.02191	−0.01902	0.074 (4)*	
H69	0.41864	0.07082	0.18963	0.139 (4)*	
H70	0.53133	0.18084	0.24663	0.139 (4)*	
H71	0.46309	0.18667	0.27036	0.139 (4)*	
H72	0.47212	0.17551	0.00362	0.139 (4)*	
H73	0.47439	0.11862	0.08686	0.139 (4)*	
H74	0.54574	0.19823	0.07020	0.139 (4)*	
H75	0.49633	0.28512	0.21228	0.139 (4)*	
H76	0.55918	0.26284	0.20982	0.139 (4)*	
H77	0.54325	0.29705	0.10563	0.139 (4)*	
H78	0.40054	0.21738	0.15621	0.139 (4)*	
H79	0.37509	0.13485	0.11920	0.139 (4)*	
H80	0.41666	0.20176	0.03683	0.139 (4)*	
H55	0.96519	0.28344	0.31579	0.0646 (13)*	
O82	0.00000	0.00000	1.127 (2)	0.1000*	0.640 (28)
O83	0.00000	0.00000	1.031 (2)	0.1000*	1.087 (25)
O84	0.6271 (4)	0.6381 (4)	0.1054 (10)	0.1000*	1.077 (10)
O85	0.00000	0.00000	0.9249 (17)	0.1000*	0.925 (24)

### Geometric parameters (Å, º)

**Table:**

O1—C13	1.420 (10)	C24—C20	1.550 (12)
O1—H48	0.823	C24—C21	1.349 (9)
O2—C18	1.308 (9)	C24—C30	1.516 (11)
O2—H57	0.735	C25—C22	1.425 (13)
O3—C20	1.183 (10)	C25—C23	1.502 (11)
O4—C23	1.281 (7)	C25—C27	1.348 (12)
O4—H60	1.261	C26—N9	1.329 (12)
O5—C21	1.334 (11)	C26—C22	1.259 (12)
O6—C27	1.284 (7)	C26—C32	1.469 (13)
O6—H60	1.283	C27—O6	1.284 (7)
O7—C30	1.256 (12)	C27—C25	1.348 (12)
O7—H55	0.979	C27—C31	1.421 (11)
N8—C16	1.441 (14)	C28—N8	1.369 (16)
N8—C28	1.369 (16)	C28—H49	1.141
N8—C29	1.404 (15)	C28—H50	1.140
N9—C26	1.329 (12)	C28—H51	1.140
N9—C34	1.660 (15)	C29—N8	1.404 (15)
N9—C35	1.250 (14)	C29—H52	1.141
N10—C30	1.408 (12)	C29—H53	1.140
N10—H61	1.110	C29—H54	1.139
N10—H62	1.109	C30—O7	1.256 (12)
N11—C33	1.409 (15)	C30—N10	1.408 (12)
N11—C36	1.456 (13)	C30—C24	1.516 (11)
N11—H69	1.110	C31—C27	1.421 (11)
C12—C13	1.515 (9)	C31—C32	1.456 (12)
C12—C14	1.498 (13)	C31—C33	1.313 (10)
C12—C16	1.520 (10)	C32—C26	1.469 (13)
C12—H41	1.139 (12)	C32—C31	1.456 (12)
C13—O1	1.420 (10)	C32—H56	1.056 (13)
C13—C12	1.515 (9)	C33—N11	1.409 (15)
C13—C18	1.625 (9)	C33—C31	1.313 (10)
C13—C20	1.703 (11)	C33—H58	1.139
C14—C12	1.498 (13)	C33—H59	1.139
C14—C15	1.544 (11)	C34—N9	1.660 (15)
C14—H42	1.139	C34—H63	1.139
C14—H43	1.140	C34—H64	1.140
C15—C14	1.544 (11)	C34—H65	1.140
C15—C17	1.501 (10)	C35—N9	1.250 (14)
C15—C19	1.413 (12)	C35—H66	1.140
C15—H44	0.906	C35—H67	1.139
C16—N8	1.441 (14)	C35—H68	1.140
C16—C12	1.520 (10)	C36—N11	1.456 (13)
C16—C21	1.577 (13)	C36—C37	1.631 (17)
C16—H45	1.140	C36—H70	1.107
C17—C15	1.501 (10)	C36—H71	1.120
C17—C18	1.398 (11)	C37—C36	1.631 (17)
C17—C23	1.480 (11)	C37—C38	1.356 (17)
C18—O2	1.308 (9)	C37—C39	1.709 (16)
C18—C13	1.625 (9)	C37—C40	1.388 (17)
C18—C17	1.398 (11)	C38—C37	1.356 (17)
C19—C15	1.413 (12)	C38—H72	1.140
C19—C22	1.663 (10)	C38—H73	1.140
C19—H46	1.140	C38—H74	1.139
C19—H47	1.139	C39—C37	1.709 (16)
C20—O3	1.183 (10)	C39—H75	1.031
C20—C13	1.703 (11)	C39—H76	1.182
C20—C24	1.550 (12)	C39—H77	1.106
C21—O5	1.334 (11)	C40—C37	1.388 (17)
C21—C16	1.577 (13)	C40—H78	1.141
C21—C24	1.349 (9)	C40—H79	1.139
C22—C19	1.663 (10)	C40—H80	1.140
C22—C25	1.425 (13)	O82—O83	1.39 (3)
C22—C26	1.259 (12)	O83—O82	1.39 (3)
C23—O4	1.281 (7)	O83—O85	1.55 (3)
C23—C17	1.480 (11)	O85—O83	1.55 (3)
C23—C25	1.502 (11)		
			
C13—O1—H48	118.0	O6—C27—C25	130.3 (11)
C18—O2—H57	90.3	O6—C27—C31	111.3 (11)
C23—O4—H60	97.8	C25—C27—C31	118.0 (8)
C27—O6—H60	94.4	N8—C28—H49	109.4
C30—O7—H55	106.5	N8—C28—H50	109.6
C16—N8—C28	110.2 (13)	H49—C28—H50	109.7
C16—N8—C29	127.3 (14)	N8—C28—H51	109.5
C28—N8—C29	111.5 (15)	H49—C28—H51	109.4
C26—N9—C35	125.6 (10)	H50—C28—H51	109.2
C30—N10—H61	109.5	N8—C29—H52	109.4
C30—N10—H62	109.5	N8—C29—H53	109.5
H61—N10—H62	109.5	H52—C29—H53	109.4
C33—N11—C36	124.0 (12)	N8—C29—H54	109.6
C33—N11—H69	109.5	H52—C29—H54	109.4
C36—N11—H69	101.7	H53—C29—H54	109.5
C13—C12—C14	107.9 (8)	O7—C30—N10	107.8 (9)
C13—C12—C16	113.3 (8)	O7—C30—C24	122.8 (11)
C14—C12—C16	104.8 (9)	N10—C30—C24	115.1 (11)
C13—C12—H41	110.2	C27—C31—C32	115.8 (10)
C14—C12—H41	110.2	C27—C31—C33	124.4 (12)
C16—C12—H41	110.2	C32—C31—C33	118.4 (11)
O1—C13—C12	108.0 (9)	C26—C32—C31	122.5 (10)
C12—C14—C15	111.3 (9)	C26—C32—H56	90.4
C12—C14—H42	109.3	C31—C32—H56	144.9 (11)
C15—C14—H42	109.1	N11—C33—C31	116.8 (13)
C12—C14—H43	109.5	N11—C33—H58	109.5
C15—C14—H43	108.4	C31—C33—H58	105.6
H42—C14—H43	109.2	N11—C33—H59	108.2
C14—C15—C17	112.1 (8)	C31—C33—H59	108.2
C14—C15—C19	117.1 (10)	H58—C33—H59	108.3
C17—C15—C19	105.7 (9)	H63—C34—H64	109.5
C14—C15—H44	86.0	H63—C34—H65	109.5
C17—C15—H44	124.9	H64—C34—H65	109.5
C19—C15—H44	110.8	N9—C35—H66	109.4
N8—C16—C12	101.5 (10)	N9—C35—H67	109.4
N8—C16—H45	110.8	H66—C35—H67	109.5
C12—C16—H45	110.8	N9—C35—H68	109.4
C15—C17—C18	122.7 (4)	H66—C35—H68	109.5
C15—C17—C23	123.4 (9)	H67—C35—H68	109.5
C18—C17—C23	112.5 (8)	N11—C36—H70	121.5
O2—C18—C17	130.4 (8)	N11—C36—H71	99.8
C15—C19—H46	109.5	H70—C36—H71	105.3
C15—C19—H47	107.7	C38—C37—C40	107.0 (16)
H46—C19—H47	107.6	C37—C38—H72	109.4
O3—C20—C24	126.0 (10)	C37—C38—H73	109.4
O5—C21—C24	119.7 (8)	H72—C38—H73	109.4
C25—C22—C26	123.5 (7)	C37—C38—H74	109.5
O4—C23—C17	121.4 (9)	H72—C38—H74	109.5
O4—C23—C25	123.3 (9)	H73—C38—H74	109.6
C17—C23—C25	113.0 (8)	H75—C39—H76	114.4
C20—C24—C21	125.5 (8)	H75—C39—H77	121.1
C20—C24—C30	112.1 (9)	H76—C39—H77	108.8
C21—C24—C30	120.3 (9)	C37—C40—H78	109.4
C22—C25—C23	123.7 (9)	C37—C40—H79	109.5
C22—C25—C27	123.8 (7)	H78—C40—H79	109.5
C23—C25—C27	111.8 (10)	C37—C40—H80	109.5
N9—C26—C22	122.7 (10)	H78—C40—H80	109.4
N9—C26—C32	121.0 (10)	H79—C40—H80	109.5
C22—C26—C32	115.9 (8)	O4—H60—O6	155.4 (5)

### Hydrogen-bond geometry (Å, º)

**Table:**

*D*—H···*A*	*D*—H	H···*A*	*D*···*A*	*D*—H···*A*
O7—H55···O3	0.98	1.84	2.614 (15)	134
O2—H57···O4	0.74	1.78	2.506 (15)	169
O6—H60···O4	1.28	1.26	2.485 (15)	155
O1—H48···O2	0.82	2.42	2.721 (13)	103
N10—H61···O5	1.11	1.79	2.686 (12)	135
N10—H62···O2^i^	1.11	2.04	2.935 (13)	135
N11—H69···O84^ii^	1.11	2.15	3.18 (2)	153
C33—H58···N10^iii^	1.14	2.51	3.63 (2)	165
C34—H64···O1^iv^	1.14	2.35	3.46 (2)	165
C35—H68···O1^iv^	1.14	2.58	3.642 (15)	154
C39—H76···O85^v^	1.18	2.41	3.32 (2)	132

Symmetry codes: (i) −*y*+4/3, *x*−*y*−1/3, *z*−1/3; (ii) −*y*+1, *x*−*y*, *z*; (iii) −*y*+2/3, *x*−*y*−2/3, *z*+1/3; (iv) −*y*+2/3, *x*−*y*−2/3, *z*−2/3; (v) *x*+2/3, *y*+1/3, *z*−2/3.
